# Untargeted Lipidomics Analysis Unravels the Different Metabolites in the Fat Body of Mated Bumblebee (*Bombus terrestris*) Queens

**DOI:** 10.3390/ijms242015408

**Published:** 2023-10-21

**Authors:** Yueqin Guo, Fugang Liu, Yulong Guo, Yingping Qu, Zhengyi Zhang, Jun Yao, Jin Xu, Jilian Li

**Affiliations:** State Key Laboratory of Resource Insects, Institute of Apicultural Research, Chinese Academy of Agricultural Sciences, Beijing 100193, China; guoyueqin@caas.cn (Y.G.); liufugangl@outlook.com (F.L.); dickbest@sina.cn (Y.G.); quyingpingq@outlook.com (Y.Q.); zhangzhengyi@mail.tsinghua.edu.cn (Z.Z.); yaojun@caas.cn (J.Y.); xujin@caas.cn (J.X.)

**Keywords:** bumblebee, *Bombus terrestris*, fat body, untargeted lipid metabolomics, differential metabolites, lipid droplet

## Abstract

The fat body has important functions in energy, fertility, and immunity. In female insects, mating stimulates physiological, behavioral, and gene expression changes. However, it remains unclear whether the metabolites in the fat body are affected after the bumblebee (*Bombus terrestris*) queen mates. Here, the ultrastructure and lipid metabolites in fat body of mated queens were compared with those of virgins. The fat body weight of mated bumblebee queens was significantly increased, and the adipocytes were filled with lipid droplets. Using LC-MS/MS-based untargeted lipidomics, 949 and 748 differential metabolites were identified in the fat body of virgin and mated bumblebee queens, respectively, in positive and negative ion modes. Most lipid metabolites were decreased, especially some biomembrane components. In order to explore the relationship between the structures of lipid droplets and metabolite accumulation, transmission electron microscopy and fluorescence microscopy were used to observe the fat body ultrastructure. The size/area of lipid droplets was larger, and the fusion of lipid droplets was increased in the mated queen’s fat body. These enlarged lipid droplets may store more energy and nutrients. The observed differences in lipid metabolites in the fat body of queens contribute to understanding the regulatory network of bumblebees post mating.

## 1. Introduction

Bumblebees and honey bees are social insects, which are essential pollinators and play important roles in natural and agricultural ecosystems [1,2,3]. About three-quarters of crops in the world depend on insect pollination, especially bee pollination, which has an obvious effect on improving crop production and fruit quality [4]. There are diverse landforms in North China, including plateaus, plains, mountains, and deserts, making it one of the most diverse districts for pollinators in the world [5]. Previous studies on the reproduction of bumblebee queens have focused on ovarian status, hormone effects, nutritional metabolism, feed composition, intestinal bacteria species, and environmental factors [6,7,8,9,10,11]. Most insects reproduce sexually, and the physiology and behavior of female insects change post mating [12,13,14,15,16,17]. After honey bee and bumblebee queens mate with drones, the expressions of immune- and sperm storage-related genes in spermathecae are significantly up-regulated [18,19]. Copulation also stimulates the activation of acquired immunity in the haemolymph of bumblebees, and the immunity is maintained continuously during diapause [20]. However, it is unclear whether there is any change in the fat body between the virgin and mated bumblebee (*B. terrestris*) queens at 7 days post mating.

The fat body of insects plays essential roles in energy storage, fertility, and immunity [21]. The fat body is mostly distributed on the insects dorsal and ventral sides, and is adjacent to the stratum corneum, similar to the liver of vertebrates [22]. The fat body is made up of adipocytes that include trophoblastic and endothelial cells [23,24]. In the bee abdomen, the fat body is an important immune tissue, and it can produce antimicrobial peptides, lysozymes and other essential proteins, which are released into the haemolymph [25]. Three major classes of macronutrients including carbohydrates, proteins, and lipids, which are required for survival and reproduction, are converted from dietary nutrients to energy and biomass in a conservative process [26]. The fat body is an important tissue involved in nutrient storage, metabolism, and immunity [21,27,28]. The fat body transmits the nutritional status of the body to the brain, activates the synthesis of insulin-like peptides (ILP), and regulates metabolism [29]. In the adipocytes of *Drosophila melanogaster*, amino acid nutrition perception and diet-regulated metabolic pathways affect the preservation of female germ line stem cells (GSCs) [30], as well as the survival and yolk formation of female germline cysts [31,32]. The expression of vitellogenin (Vg) in the fat body of bumblebees is positively correlated with their ovarian activation status [33]. Vg is synthesized in the fat body, and transported to the ovary, promoting the development of the ovary and egg [33]. In *Apis cerana* worker bees, Vg is expressed and functions as an antimicrobial and antioxidant agent in the fat body and venom gland [34]. Before the bumblebee (*B. terrestris*) queens enter diapause, a large amount of glycogen and lipid substances are stored in the fat body for metabolism during diapause [35]. At the end of a bumblebee colony, the newly emerged queens leave the nest to mate with drones. After a few days, the queens enter into diapause [8,36]. During this period, the bumblebee queens have to face unknown environmental changes, and the nutrient and energy stored in the fat body provide efficient substrates which queens need [35]. 

Lipid metabolism has important function(s) in the development and reproduction of an organism [37,38], especially in providing the required energy during long non-feeding periods [28]. A large amount of glycogen and triglycerides (TAG) are stored in the fat body [21]. In the fat body, lipids are preserved in lipid droplets in the cytoplasm, with TAG being the main component of lipid [21,28]. Lipids account for 30–40% of the dry egg’s weight, most of which is TAG, and these lipids are the main energy for embryonic development [39]. Insect oocytes synthesize TAG from free fatty acids (FFA) and glycerol, but the required fatty acids (FA) must be obtained from the fat body or diet [39]. Diacylglycerol (DAG), TAG, and FA can be converted to each other in multiple tissues [38]. There are molecules in the fat body of bumblebee (*Bombus impatiens*) that react to the sucrose level in food [35], and when queens are treated with CO_2_, the lipid content in the fat body decreases, while the glycogen and protein content in the ovary increases, indicating that CO_2_ promotes the transfer of a large number of nutrients and the utilization of storage reserves by accelerating metabolism [36]. EGFR signaling in the fat body of honey bees (*Apis mellifera*) is associated with an increase in body size and a shortened developmental time under the influence of royal jelly or royalactin [40]. These phenomena reveal that there are response factors in the fat body to food and environmental stimulate. The fat body of bumblebee (*B. terrestris*) queens stores and provides nutrients such as glycogen and lipids [35]. Therefore, the fat body is essential for the healthy survival and ovarian status of bumblebee queens. Although the fat body has important function(s) in various physiological processes in insects, it remains unclear what occurs in the fat body after the bumblebee queens mate.

Metabolomics can be used to identify the small molecule metabolites (<1500 Da) in organisms, and the abundance of metabolites is detected and analyzed from a holistic perspective [41]. The metabolomics techniques can be separated into untargeted and targeted metabolomics. Untargeted metabolomics techniques are widely used, with the benefits of high throughput, panoramic analysis, and technical flexibility [42]. LC-MS/MS-based untargeted metabolomics techniques were used to analyze the metabolic alteration in the fat body of *Galleria mellonella* larvae [43]. Using untargeted metabolomics techniques, the apoptotic brown adipocytes were observed to discharge a particular set of metabolites in which purine metabolites are highly enriched [44]. 

To date, no reports have focused on the metabolites contained in the fat body of bumblebee queens, although the fat body has important functions in bumblebee reproduction. In this study, we focused on the ultrastructure and metabolomics differences in the fat body of virgin (V) and mated (M) bumblebee queens at 7 days post mating. The fat body weight of mated queens was significantly increased at 7 days post mating, compared with the virgins. In the fat body of mated bumblebee queens, the adipocytes were filled with lipid droplets. The untargeted lipid metabolomics results showed that many kinds of metabolites were significantly decreased in the fat body of mated queens compared with the fat body of the virgins. Many kinds of PE and PC isomers were significantly decreased in the fat body of queens post mating, and these lipid metabolites were the important components of biomembrane. Consistently, the size of lipid droplets and the fusion of lipid droplets were significantly increased in the fat body of queens post mating compared with the virgins. Thus, our data reveal that copulation stimulated lipid metabolite changes in the fat body of bumblebee queens.

## 2. Results

### 2.1. An Obvious Increase in the Fat Body Weight of Mated Queens

**Figure 1:**
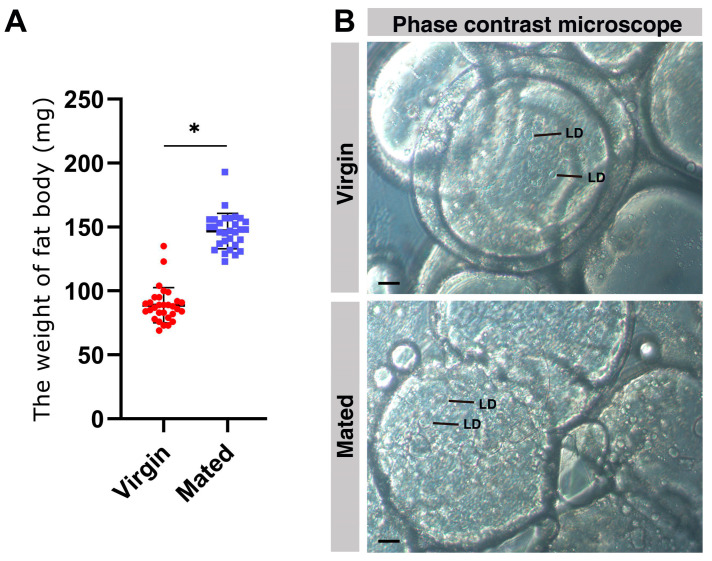
The weight and phase contrast microscope images of virgin and mated bumblebee queen fat bodies. (**A**) The fat body weight of mated queens (blue points, 146.9 ± 13.86 mg, *n* = 30) at 7 days post mating was significantly increased compared with that of the virgins (red points, 88.93 ± 13.75 mg, *n* = 30). Values represent mean ± SD. * *p* < 0.05. (**B**) Phase contrast microscope images of the fat bodies of virgin and mated bumblebee queens at 7 days post mating. The adipocytes were filled with lipid droplets in the fat body of mated bumblebee queens at 7 days post mating, and there were significantly fewer lipid droplets in cells of virgin queens than the mated queen cells. LD: lipid droplet; Scale bars, 10 µm.

Here, we compared the weight of the fat body in virgin and mated bumblebee queens at 7 days post mating. The data show that the fat body weight of mated bumblebee (*B. terrestris*) queens was significantly increased compared with the virgins (Mated: 146.9 ± 13.86 mg, Virgin, 88.93 ± 13.75 mg, * *p* < 0.05.) (Figure 1A). Then, the microstructure of the fat body was observed with a phase contrast microscope. The adipocytes were filled with lipid droplets iIn the fat body of mated bumblebee queens at 7 days post mating, and there were clearly fewer lipid droplets in the cells of virgin queens than in mated queen cells (Figure 1B). 

### 2.2. Metabolic Profiling of the Fat Bodies of Virgin and Mated Bumblebee Queens

In order to examine whether lipid metabolites in the fat body were affected, lipidomic profiling of the fat bodies of virgin and mated bumblebee (*B. terrestris*) queens was performed by untargeted lipid metabolomics. The metabolome of virgin and mated bumblebee queen fat bodies was measured using LC-MS/MS. The total ion current (TIC) of the QC samples were superimposed and compared in the positive and negative ion detection modes. Observing the results, the overlap degree of each chromatographic peak was large, and the shape of the TIC peak was entire. The separation between adjacent peaks was obvious, which indicates that the detection system was steady and dependable in the negative and positive ion mode. The heat map of hierarchical clustering analysis (HCA) in positive and negative ion modes across all samples is provided in Supplementary Materials (Figure S1). 

### 2.3. Metabolite Analysis and Differential Accumulation of Metabolites 

**Figure 2:**
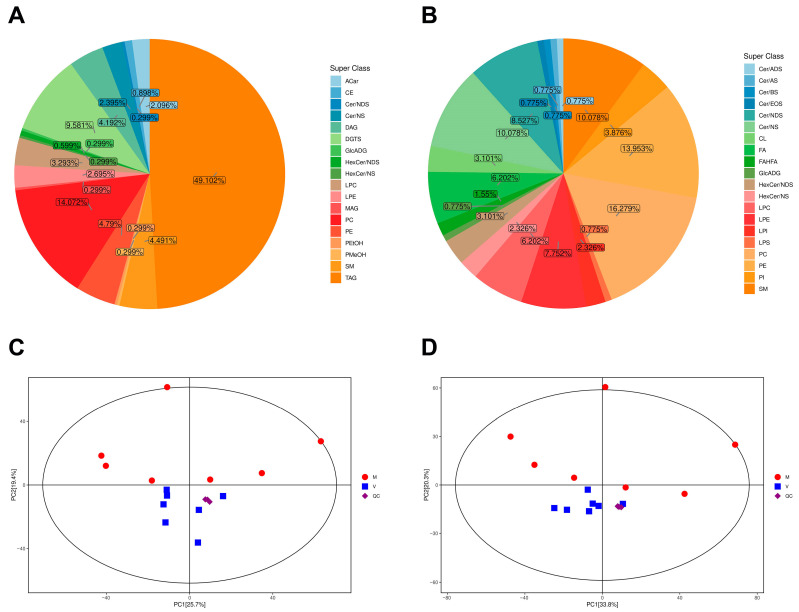
(**A**) Pie chart of the percentage of each superclass of metabolites in the positive ion modes. (**B**) Pie chart of the percentage of each superclass of metabolites in the negative ion modes. (**C**) PCA analysis of all samples based on peaks observed in the positive ion modes. (**D**) PCA analysis of all samples based on peaks observed in the negative ion modes.

The identified metabolites were classified and quantified according to the available data on chemical classification. In the positive ion mode, 334 metabolites were detected in total and could be categorized into 18 different classes, including 164 triacylglycerols (TAGs), 47 phosphatidylcholines (PCs), 32 diacylglyceryl trimethylhomoserines (DGTSs), 16 phosphatidylethanolamines (PEs), 15 sphingomyelins (SMs), 14 diacylglycerols (DAGs), 11 lysophophatidylcholines (LPCs), 9 lysophosphatidylethanolamines (LPEs), 8 ceramide non-hydroxyfatty acid-sphingosines (Cer-NSs), 7 acylcarnitines (ACars), 3 cholesteryl esters (CEs), 2 hexosylceramide non-hydroxyfatty acid-dihydrosphingosines (HexCer-NDSs), 1 ceramide non-hydroxyfatty acid-dihydrosphingosine (CER-NDS), 1 glucuronosyldiacylglycerol (GlcADG), 1 hexosylceramide non-hydroxyfatty acid-sphingosine (HexCer-NS), 1 monoacylglycerol (MAG), 1 phosphatidylethanol (PEtOH), and 1 phosphatidylmethanol (PMeOH) (Figure 2A). Among these, TAGs were the largest class of metabolites in the positive ion mode (Figure 2A). 

A total of 129 metabolites were detected in the negative ion mode and could be categorized into 19 different classes, including 21 PCs, 18 PEs, 13 SMs and Cer-NSs, 11 Cer-NDSs, 10 LPEs, 8 LPCs and Free fatty acids (FAs), 5 Phosphatidylinositols (PIs), 4 HexCer-NDSs and cardiolipins (CLs), 3 lysophosphatidylinositols (LPIs) and HexCer-NSs, 2 fatty acid ester of hydroxyl fatty acids (FAHFAs), 1 ceramide alpha-hydroxy fatty acid-dihydrosphingosine (Cer-ADS), 1 ceramide alpha-hydroxy fatty acid-sphingosine (Cer-AS), 1 ceramide beta-hydroxy fatty acid-sphingosine (Cer-BS), 1 ceramide esterified omega-hydroxy fatty acid-sphingosine (Cer-EOS), 1 GlcADG, and 1 lysophosphatidylserine (LPS) (Figure 2B). Among these, PCs were the largest class of metabolites in the negative ion mode (Figure 2B).

### 2.4. Principal Component Analysis of Differential Metabolites

Principal component analysis (PCA) was performed to visualize the overall metabolic differences and relationships among samples. The metabolite profiles of the 14 samples (2 different collecting groups × 7 replicates) in the positive and negative ion detection modes were generated, which demonstrated that the two groups could be identified in the PC1 × PC2 score plots (Figure 2C,D). The PCA of all samples (including QC samples) showed variation within two groups (Figure 2C,D). 

At the first principal component, there is an obvious separation between the virgin and mated bumblebee queens at 7 days post mating, and the samples of the same group were gathered together, indicating the stability of system and the reliability of the collected data. The first principal component (PC1) explained 25.7% and 33.8% of the total variation in the positive and negative ion detection modes, thus separating the two contrasting groups of virgin and mated bumblebee queens unambiguously. The separation between the virgin and mated groups indicated the metabolism of fat body was significantly affected in the fat body of mated queens at 7 days post mating. In general, our experimental design and data are solid and suitable for downstream analysis. 

### 2.5. Orthogonal Partial Least Squares Discriminant Analysis of Differential Metabolites

To identify differential metabolites in the fat body of virgin and mated queens, orthogonal partial least squares discriminant analysis (OPLS−DA) was performed in two comparison groups, and variable importance in the projection (VIP) values were obtained. OPLS is a supervised orthogonal partial least squares method, and OPLS regression is a stoichiometric projection method which correlates the X and Y variables through a linear multivariate model. They are commonly used to study metabolic profiles and identify different metabolites [42,45]. The quality of the OPLS regression model is verified by a correlation coefficient R^2^ (goodness to fit) and a cross-validated correlation coefficient Q^2^ (goodness of prediction) [42,46]. 

**Figure 3:**
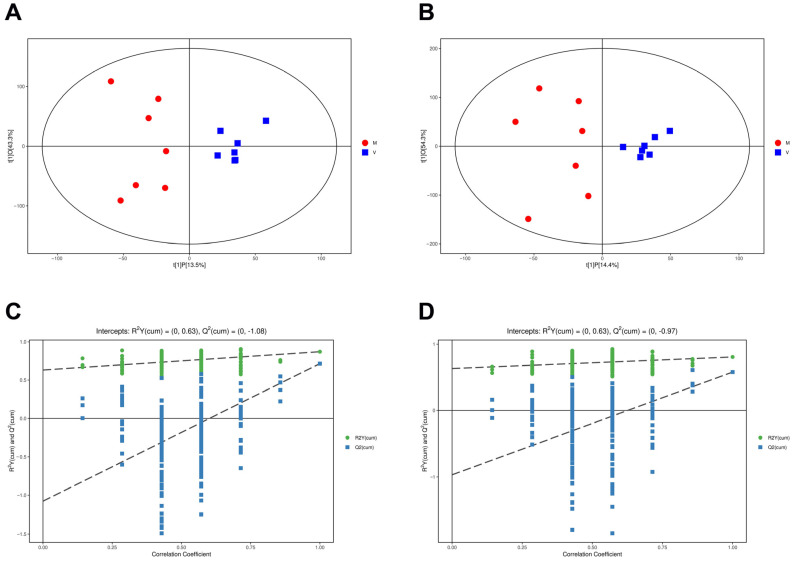
OPLS-DA analysis of untargeted metabolomics data. (**A**) The scatter plot of OPLS regression score in the positive ion mode. (**B**) The scatter plot of OPLS regression scores in the negative ion mode. (**C**) Alignment test of OPLS-DA model in the positive ion mode. (**D**) Alignment test of OPLS-DA model in the negative ion mode.

Two OPLS regression models were established in the current study to regress differential metabolite abundance against time in positive and negative ion modes. The OPLS model illustrated fine repeatability of examples in the same group and an obvious separation degree between the two groups, so we applied the OPLS model to investigate the distribution of the samples (Figure 3A,B). 

In Figure 3C,D, the horizontal coordinates represent the displacement retention degree of the displacement test, the vertical coordinates represent the R^2^Y or Q^2^ value of the original model, the green dot represents the R^2^Y value obtained by the displacement test, and the blue square dot represents the Q^2^ value obtained by the displacement test. The two dotted lines represent the regression lines of R^2^Y and Q^2^, respectively. The R^2^Y and Q^2^ values in the positive ion mode were 0.63 and −1.08, and the R^2^Y and Q^2^ values in the negative ion mode were 0.63 and -0.97, respectively (Figure 3C,D). When the slopes of the two regression lines are positive, we consider the model of the OPLS-DA diagram to be reliable.

### 2.6. Screening of Differential Metabolites

**Figure 4:**
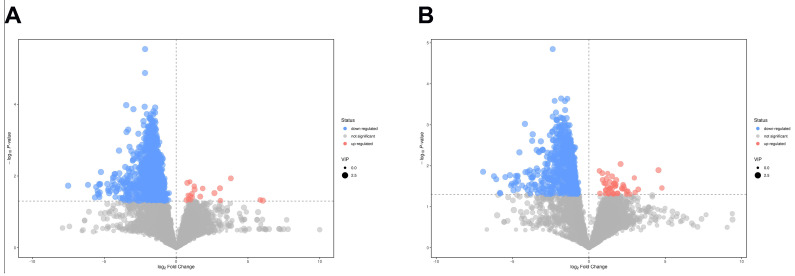
Volcano plots of the increased and decreased metabolites in the fat body of mated bumblebee queens, compared with virgins. (**A**) In the positive ion mode. (**B**) In the negative ion mode. The red dots indicate the significantly increased metabolites, blue dots indicate the significantly decreased metabolites (*p* < 0.05 and VIP > 1), and the gray dots indicate no significance in the fat body of mated bumblebee queens compared with the virgins.

Based on the screening criteria of VIP > 1 and *p* < 0.05, from all the detected metabolites in the fat body of the virgin and mated bumblebee queens, a total of 949 differential metabolites were investigated in the positive ion detection modes, among which 19 differential metabolites were increased, and 930 differential metabolites were decreased. There were total of 748 differential metabolites in the negative ion detection modes, among which 47 differential metabolites were increased, and 701 differential metabolites were decreased. The differential metabolites of different comparisons in the positive and negative ion mode were visualized using volcano plots (Figure 4A,B). 

### 2.7. Hierarchical Cluster Analysis of Differential Metabolites

The differential metabolites screened often have similar or complementary results and functions in biology, may be positively or negatively regulated by the same metabolic pathway, and exhibit similar or opposite expression characteristics between differential groups. The HCA of these characteristics categorizes metabolites with the same characteristics into one group, and reveals how the characteristics of the metabolites differ between experimental groups. The Euclidean distance matrix for the quantitative values of differential metabolites was calculated. The differential metabolites were clustered by complete linkage method and displayed as heatmaps.

**Figure 5:**
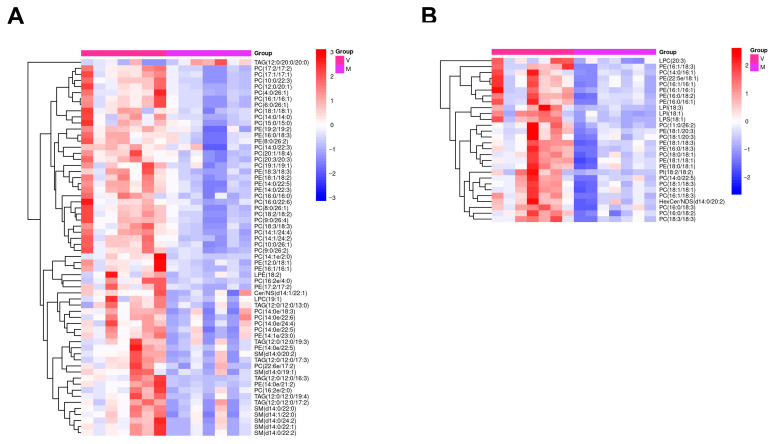
Heatmaps of differential metabolites in the fat bodies of virgin and mated bumblebee queens. (**A**) In the positive ion mode. (**B**) In the negative ion mode. Differential metabolites are identified based on VIP > 1 and *p* < 0.05 for the comparison groups in the fat body.

The heatmaps of the HCA in positive and negative ion modes across all samples are shown (Figure 5). In the fat body of mated bumblebee queens, the levels of PC(17:2/17:2), PC(17:1/17:1), PC(10:0/22:3), PC(12:0/20:1), PC(4:0/26:1), PC(16:1/16:1), PC(6:0/26:1), PC(18:1/18:1), PC(14:0/14:0), PC(15:0/15:0), PE(19:2/19:2), PE(16:0/18:3), PE(8:0/26:2), LPC(20:3), PE(16:1/18:3), PC(14:0/16:1), PE(22:5e/18:1), PC(16:1/16:1), PE(16:1/16:1), PE(16:0/18:2), PE(16:0/16:1), LPI(18:3), LPI(18:1), LPS(18:1), PC(11:0/26:2), PE(18:1/20:3), and PC(18:1/20:3) were significantly decreased (Figure S2), compared with levels in the fat body of the virgins. Some of these metabolites, such as PCs and PEs, are important components of biomembranes [47,48]. 

### 2.8. ROC Analysis of Metabolites

The differential ability of metabolites is evaluated by the ROC curve. The performance of metabolites is assessed by the area under the ROC curve (AUC), and the higher the AUC value, the better performance of the metabolites. In general, the accuracy is low when the AUC is between 0.5 and 0.7; the accuracy is good if the AUC value is between 0.7 and 0.9; and if the AUC value is above 0.9, the accuracy is excellent [49]. 

The ROC curves for the 28 metabolites were constructed (Figure S3). Applying the best cut-off value for each ROC curve, the AUC value was calculated, indicating high accuracy of the 28 metabolites in the fat body of mated bumblebee queens at 7 days post mating (Figure S3). 

### 2.9. The Fusion of Lipid Droplets in the Fat Body of Mated Bumblebee Queens

**Figure 6:**
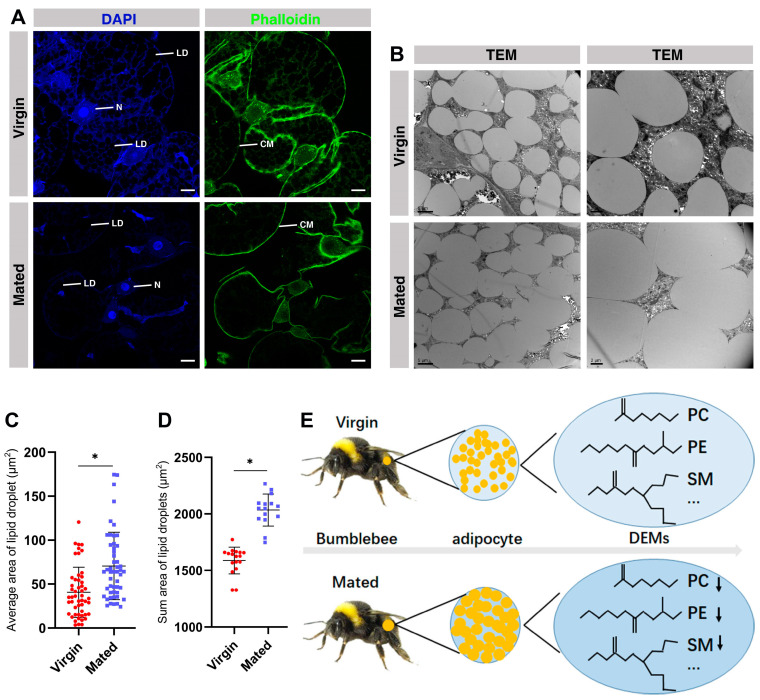
The size of lipid droplets and fusion of lipid droplets are significantly increased in the fat body of bumblebee queens post mating. (**A**) Confocal images of 40× lens/1.0 zoom in the fat body of virgin and mated bumblebee queens at 7 days post mating are shown. In all the panels except graphs, blue indicates DAPI staining, and green indicates Phalloidin staining. LD: lipid droplet; N: nucleus; Scale bars, 25 µm. (**B**) TEM images from the fat body of virgin and mated bumblebee queens at 7 days post mating. The size of lipid droplets and fusion of lipid droplets are significantly increased in the fat body of bumblebee queens at 7 days post mating compared with the virgins. Scale bars left, 5 µm. Scale bar right, 2 µm. (**C**) Quantification of the lipid droplet areas in the fat body of virgin and mated bumblebee queens in TEM (Virgin, red points, 40.66 ± 28.58 μm^2^; Mated, blue points, 70.65 ± 38.34 μm^2^). Values represent mean ± SD. Significant differences were determined by one way ANOVA/Dunn’s Method. Asterisks indicate significant differences between the fat body of Virgin and Mated. * *p* < 0.05. (**D**) Quantification of the lipid droplet sum areas in the fat body of virgin and mated bumblebee queens in TEM (Virgin, red points, 1589 ± 118.6 μm^2^; Mated, blue points, 2035 ± 141.3 μm^2^). Values represent mean ± SD. Significant differences were determined by one way ANOVA/Dunn’s Method. Asterisks indicate significant differences between the fat body of Virgin and Mated. * *p* < 0.05. (**E**) In the fat body of mated bumblebee (*B. terrestris*) queens, the adipocytes were filled with lipid droplets, and the fusion of lipid droplets was significantly increased. Most of lipid metabolites, especially some biomembrane components, such as PCs, PEs and SMs, were significantly decreased in the fat body of mated bumblebee queens. The arrows indicate the decreased metabolites, for example, PC, PE, and SM.

To further confirm this finding, fluorescence microscopy and TEM analysis were performed on the fat body of virgin and mated bumblebee queens at 7 days post mating. The confocal and TEM data show that the membrane of lipid droplets was fused in the mated bumblebee queens (Figure 6A,B). The TEM images show that the areas/sizes of lipid droplets in the fat body of mated queens were clearly increased, compared with those in virgins (Virgin, 40.66 ± 28.58 μm^2^; Mated, 70.65 ± 38.34 μm^2^, * *p* < 0.05.) (Figure 6B,C). The sum of areas/sizes of lipid droplets in the fat body of mated queens was clearly increased, compared with virgins (Virgin, 1589 ± 118.6 μm^2^; Mated, 2035 ± 141.3 μm^2^, * *p* < 0.05.) (Figure 6B,D). These results demonstrate that the membrane fusion of lipid droplets in the mated bumblebee queens was consistent with a decrease in some biomembrane components, such as PCs and PEs. 

The fat body has important functions in energy storage, fertility, and immunity. Female insects mating stimulates substantial physiological, behavioral, and gene expression changes. The adipocytes were filled with lipid droplets in the fat body of mated bumblebee (*B. terrestris*) queens. Based on the untargeted lipidomics, most of the lipid metabolites were significantly decreased in the fat body of mated bumblebee queens, especially some biomembrane components, such as PCs, PEs, and SMs. The size/area of lipid droplets was larger, and the fusion of lipid droplets was significantly increased in the fat body of mated bumblebee queens (Figure 6E). The differences in lipid metabolites in the fat body of queens contribute to understanding the regulatory network of bumblebees post mating.

## 3. Discussion

In this study, untargeted lipidomics profiling was firstly used to provide a global, high-throughput picture of the lipid metabolites in the fat body of virgin and mated bumblebee (*B. terrestris*) queens at 7 days post mating. The aim was to begin to address the molecular mechanisms by which bumblebee queens store energy and nutrient in the fat body post mating.

The fat body in bumblebees (*B. terrestris*) is responsive to the social context, and antimicrobial effector genes are expressed in the fat body [22]. In silkworm *Bombyx mori*, starvation can activate antimicrobial peptides (AMPs) gene expression in the fat body via the insulin-like signaling (ILS)/FoxO signaling pathway [50]. In honey bees (a mixture of *Apis mellifera ligustica* and *Apis mellifera*), mild winters are associated with a lower investment in glycerol synthesis and an increased expression of fat body genes, notably apidaecin and vitellogenin [51]. In the fat body of *Drosophila*, the immune system impairs growth and nutrient storage by reducing insulin signaling [52]. 

The fat body plays important roles in the life of insects, and is an important organ involving in the processing, storage, and utilization of energy and nutrients [21]. Here, our results show that the fat body weight of mated queens was significantly increased compared with the virgins (Figure 1A). In the fat body of mated bumblebee queens, the adipocytes were filled with lipid droplets (Figure 1B). In recent years, lipid droplets have emerged as crucial organelles with key functions in lipid and energy balance [53,54]. The results in this study indicate that the nutrient substrate and energy may be accumulated in the fat body of mated bumblebee queens at 7 days post mating. The bumblebee queens enter diapause a few days post mating, and the accumulation of nutrition in the fat body can provide more nutrient and energy for the queens. Adipocytes, the main fat body cells, maintain glycogen and triglycerides as energy reserves. Lipid metabolism is necessary for organism growth and reproduction, and provides the energy required during non-feeding periods [28]. Changes in the fat body of virgin and mated bumblebees were observed by high-throughput untargeted lipidomics profiling, and the results demonstrate that our methodology correctly reveals the metabolic basis of mating. Totals of 949 and 748 differential metabolites were identified in the positive and negative ion modes, respectively. Among them, TAGs and PCs were the largest class of metabolites in the positive and negative ion modes (Figure 2A,B). TAGs are the most important source of calories for maintaining energy homeostasis. Moreover, TAG conversion is essential for the metabolism of structural and signaling lipids [38]. TAGs are aggregated in the fat body of obese flies, an organ similar to mammalian liver, which specializes in lipid preservation and catabolism [55]. Roughly 30–40% of the dry weight of an insect egg consists of lipids, mostly TAGs, which are essential for the developing embryo [39]. Certain kinds of lipid metabolites were significantly decreased in the fat body of bumblebee queens at 7 days post mating, especially the components of biomembranes, such as PEs, PCs and SMs. In the membrane composition of *Diamesa tonsa* and *Pseudodiamesa branickii*, the main membrane lipids are PEs, and the high PEs/PCs ratio may enable the formation of highly deformable membranes, which increases their frost resistance [56]. The major phospholipids of Sf9 cells are PEs and PCs [57]. The decrease in these metabolites may provide energy and nutrients for other organs of mated queens, for example, the spermathecae storing sperm. The above differential metabolites might be important contributors to the differences between the virgin and mated bumblebee queens. These differential metabolites may have similar or complementary effects and functions in biology, so the decrease in these differential metabolites influence queens after mating.

To further explore the relationship between the structure of lipid droplets and metabolite changes in the fat body of queens, TEM and conjoint metabolites analyses were conducted for the comparison of virgin and mated bumblebee queens at 7 days post mating. Eukaryotic cells preserve neutral lipids in cytoplasmic lipid droplets, which are encased in a monolayer of associated proteins and phospholipids. These dynamic organelles are the main reservoirs for reserving cellular energy and forming membrane lipids [58]. Intracellular lipid droplets provide fatty acids for energy, lipoprotein secretion and membrane biogenesis [59]. Insect adipocytes conserve large quantities of lipids in the form of cytoplasmic lipid droplets [28]. In the fat body of mated bumblebee queens, the size of lipid droplets were obviously increased, and the membranes of the lipid droplets were fused, which is consistent with the decrease in biomembrane components, such as PCs, PEs, SMs, LPCs etc. Lipid droplet fusion is an important mechanism of lipid droplet growth and fat storage [60,61]. The fusion of lipid droplets promotes effective TAG storage [53]. The surface monolayer of lipid droplets is composed of phospholipids, primarily PCs, which stabilize the neutral lipid core of TAGs [59]. By combining the metabolomics and TEM data, we identified that the decreases in components in the membrane of lipid droplets occurred alongside fusion of lipid droplets in the fat body of mated bumblebee queens, which may result in more energy and nutrients in the fat body of the bumblebee queens for sperm storage and reproduction. The components of biomembranes, which were decreased in the fat body of mated queens, may be transferred to other organs. The levels of lipid metabolites changed and the lipid droplets were fused in the fat body of bumblebee queens at 7 days post mating, but the mechanism needs further in-depth research. 

Altogether, this is the first research to describe lipid metabolites in the fat body of bumblebee (*B. terrestris*) queens and reveal the changes in lipid metabolite levels after mating. The nutrition and energy reserves in the fat body have important functions in the reproduction of bumblebee queens. Further functional research on the differential metabolites should help to identify metabolites that are involved in reproduction in the fat body after queen mating and could potentially help to elucidate how these metabolites can affect bumblebee queen mating.

## 4. Materials and Methods

### 4.1. Samples Collection

Bumblebee (*B. terrestris*) queens were collected from the rearing room at the Institute of Apicultural Research, Chinese Academy of Agricultural Sciences, Beijing, China. The bumblebees were raised in an artificial breeding room (in constant darkness with a temperature of 28 ± 0.5 °C and 60 ± 5% relative humidity) and fed fresh frozen pollen and a 50% sugar solution every other day [62]. The newly emerged queens were collected from thirty independent colonies and raised in clean cages. When the virgin queens were 6 days old, these queens were randomly divided into two groups, and one group was mated with the drones, which were 10–15 days old. The copulation was accomplished in flight cages (95 × 95 × 95 cm^3^). The virgin and mated queens were kept at room temperature for 7 days before dissection for the collection of the fat body. The weight of the fat body was measured using a one over ten-thousand analytical balance (JA2003B, Shanghai, China), and 30 individual samples were obtained from virgin and mated queens. For the untargeted lipidomics profiling, 7 individual samples were obtained each from the virgin and mated queen groups. The queens were dissected from the abdomen. The epidermal layer was cut at the middle of the queens’ abdomen with a scissor, and the epidermis was fixed with 5 pins. Then, the tissues including the honey crop, midgut, posterior gut, Malpighian tubule, ovary and spermatheca were removed. (We ensured the integrity of the honey crop to avoid the leakage of honey affecting the experiment results.) The fat body was collected together with wide-headed tweezer, transferred into a clean EP tube, and frozen in liquid nitrogen immediately. All samples were stored at −80 °C until used for further experiments. 

To determine the weight of the fat body, 30 individual samples were obtained from the virgin and mated queens. The weight of the fat body was measured using an analytical balance. Statistical analysis of the fat body weight was performed with one way ANOVA/Dunn’s Method, and the data are presented as mean ± SD. The graphs were created with GraphPad Prism (Version 8), and further modified using Adobe Illustrator (Version CS6).

### 4.2. Metabolites Extraction

The fat body samples from 14 individuals (7 samples for the virgin queens, and 7 samples for the mated ones) were submitted to Biotree (Shanghai Biotree biomedical technology Co., Ltd., Shanghai, China) for untargeted lipidomics profiling. A 25 mg wet weight sample was added to an EP tube. Then, 200 μL of water, and 480 μL of extract solution (methyl tert-butyl ether (MTBE):methanol (MeOH) = 5:1, *v*/*v*) were added sequentially. After 30 s of vortexing, the samples were ground with a grinding machine at 35 Hz for 4 min and sonicated for 5 min in an ice-water bath. The grinding treatment and sonication cycle were repeated 3 times in total. The samples were subsequently incubated at −40 °C for 1 h and centrifuged at 3000× *g* for 15 min at 4 °C. Then, 300 μL of supernatant was removed to a fresh tube and dried in a vacuum concentrator at 37 °C. Then, the dried samples were reconstituted in 100 μL of 50% methanol in dichloromethane by sonication for 10 min in an ice-water bath. The reconstituted samples were then centrifuged at 13,000× *g* for 15 min at 4 °C, and 75 μL of supernatant was removed to a fresh glass vial for further LC/MS analysis. The quality control (QC) sample was prepared by mixing an equal aliquot 20 μL of the supernatants from all of the samples. 

### 4.3. LC-MS/MS Analysis

The LC-MS/MS analyses were performed with an Ultra High-Performance Liquid Chromatography (UHPLC) system (1290, Agilent Technologies, Palo Alto, CA, USA), provided with a Kinetex C18 column (2.1 × 100 mm^2^, 1.7 μm, Phenomen). The mobile phase A was composed of 40% water, 60% acetonitrile, and 10 mM ammonium formate. The mobile phase B was composed of 90% isopropanol and 10% acetonitrile, which was appended with 50 mL 10 mM ammonium formate for every 1000 mL mixed solvent. The analysis was performed with an elution gradient as follows: 0–1.0 min, 40% B; 1.0–12.0 min, 40–100% B; 12.0–13.5 min, 100% B; 13.5–13.7 min, 100–40% B; and 13.7–18.0 min, 40% B. The column temperature was set at 55 °C, and the auto-sampler temperature was set at 4 °C. The injection volume was 2 μL (positive) or 2 μL (negative). The QE mass spectrometer was applied with its capability to obtain MS/MS spectra on data-dependent acquisition (DDA) mode in the control of the acquisition software (Xcalibur 4.0.27, Thermo Fisher Corp., Waltham, MA, USA). In this mode, the acquisition software constantly evaluates the full scan MS spectrum. The electrospray ionization (ESI) source conditions were set as follows: sheath gas flow rate at 30 Arb, Aux gas flow rate at 10 Arb, capillary temperature at 320 °C (positive) or 300 °C (negative), full MS resolution at 70,000, MS/MS resolution at 17,500, collision energy at 15/30/45 in NCE mode, and spray Voltage at 5 kV (positive) or −4.5 kV (negative). 

### 4.4. Metabolome Data Preprocessing and Annotation

The raw data files were converted to files of mzXML format with the ‘msconvert’ program from ProteoWizard. The CentWave algorithm in XCMS was employed for peak observation, extraction, alignment, and integration; the minfrac and cutoff for annotation were set at 0.5 and 0.3, respectively. Lipid identification was obtained through a spectral match with LipidBlast library [63], which was performed by R and based on XCMS. 

### 4.5. Phase Contrast Microscopy

The fat body of virgin and mated bumblebee queens to be used for phase contrast microscopy was dissected in 1 × PBS (10 mM NaH_2_PO4/Na_2_HPO4, 175 mM NaCl, pH 7.4, Solarbio, Beijing, China). Immediately, the samples were fixed in 4% paraformaldehyde (Solarbio, Beijing, China) for 25 min at room temperature, washed with 0.1% Triton X-100 in 1 × PBS (1 × PBT) for 2 × 5 min, and then mounted in mounting medium. The phase contrast images were performed using a Zeiss Axio Imager A1 phase contrast microscope (Zeiss, Oberkochen, Germany), and all images were processed with Adobe Photoshop (Version CS6) and Adobe Illustrator (Version CS6).

### 4.6. Fluorescence Microscopy 

The virgin and mated bumblebee queens were dissected in 1 × PBS, and the fat body was fixed in 4% paraformaldehyde for 25 min at room temperature. The samples were rinsed, and washed with 0.1% Triton X-100 in 1 × PBS (1 × PBT) for 2 × 5 min. DAPI (Sigma-Aldrich, St. Louis, MO, USA, D9542, 1 μg/mL) and Alexa Fluor^®^ 488 Phalloidin (CST, Boston, MA, USA, 8878S, 1:1000) were added to the samples and the samples were then incubated for 1 h at room temperature. Nuclei were labeled with DAPI, and actin was specifically labeled by fluorescent phalloidin. Then, the samples were rinsed and washed with 0.1% 1 × PBT for 3 × 5 min. The samples were mounted in mounting medium [70% glycerol containing 2.5% DABCO (Sigma-Aldrich, St. Louis, MO, USA, D27802)]. Confocal fluorescence imaging was performed with a Leica SP8 laser scanning microscope (Leica, Wetzlar, Germany), and all images were processed with Adobe Photoshop (Version CS6) and Adobe Illustrator (Version CS6). 

### 4.7. Transmission Electron Microscopy (TEM)

For TEM analysis, the bumblebee queens were dissected and the fat body was immediately fixed in 2.5% glutaraldehyde in PBS for 24 h. The tissues were washed in PBS, then post-fixed in 1% osmium tetroxide. Then, the samples were washed in PBS. The graded alcohol series were subsequently used for dehydration. Then, the fat body was embedded in Epon-812 resin (Sigma-Aldrich, 45,345, St. Louis, MO, USA). Ultrathin sections (80–100 nm) were cut by an ultramicrotome (Leica M80, Wetzlar, Germany), and stained with uranyl acetate and lead citrate. Images were obtained on a transmission electron microscope (Hitachi H-7500, Tokyo, Japan) in the Institute of Food Science and Technology, CAAS. All TEM images were processed with Adobe Photoshop (Version CS6) and Adobe Illustrator (Version CS6). 

To determine the area/size of the lipid droplets and the sums of the lipid droplet area/size in fields, the TEM images from the fat body of virgin and mated queens were obtained. The images were captured in six random fields of samples, and the statistical data were obtained from more than ten samples. The area/size of the lipid droplets and the sums of the lipid droplet area/size in fields were measured with Image Pro Plus software (Version 6.0.0.260). Statistical analysis of the single lipid droplet area and the sums of lipid droplet area in fields was carried out by one way ANOVA/Dunn’s Method, and the data are reported as mean ± SD. The graphs were created with GraphPad Prism (Version 8), and further modified with Adobe Illustrator (Version CS6). 

## 5. Conclusions

In the fat body of mated bumblebee (*B. terrestris*) queens, the adipocytes were filled with lipid droplets, and the fusion of lipid droplets was significantly increased. Most of the lipid metabolites, especially some biomembrane components, were significantly decreased. 

## Data Availability

Not applicable.

## Supplementary Materials

The following supporting information can be downloaded at: https://www.mdpi.com/article/10.3390/ijms242015408/s1.
