# Supplementary material for: Untargeted Lipidomics Analysis Unravels the Different Metabolites in the Fat Body of Mated Bumblebee (Bombus terrestris) Queens
Source: Int J Mol Sci. 2023 Oct 21;24(20):15408. doi: 10.3390/ijms242015408 (PMC10607666; doi:10.3390/ijms242015408)
Supplement: Supplementary file 1 [file ijms-24-15408-s001.zip › ijms-2623546-supplementary.pdf]

International Journal of
Molecular Sciences

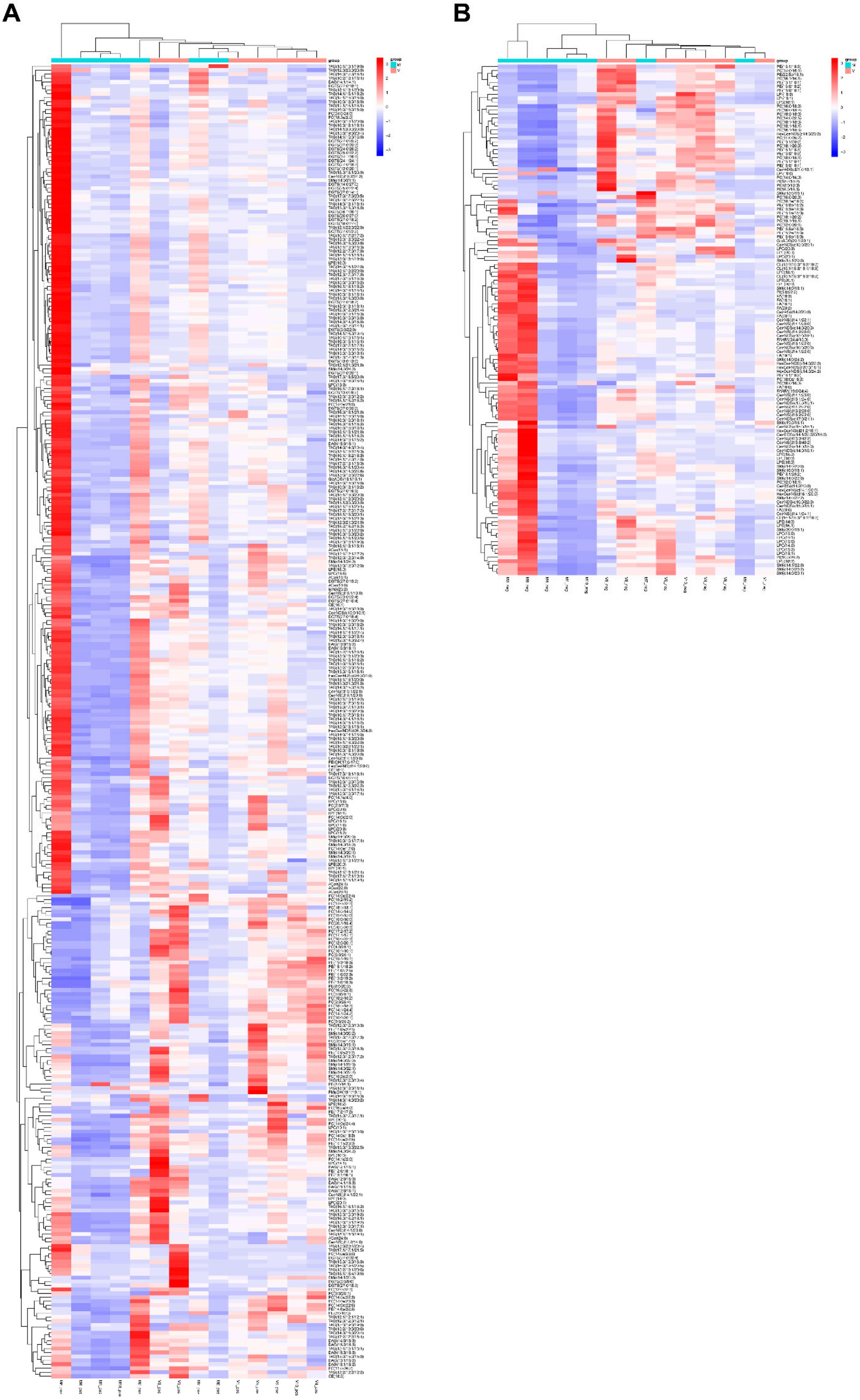

Figure S1. Heat map of the HCA of the differential metabolites in the comparison of virgin and mated in the bumblebee (*B. terrestris*). (**A**) Differential metabolites were separated using hierarchical clustering in the positive ion detection mode. (**B**) Differential metabolites were separated using hierarchical clustering in the negative ion detection mode. The x-axis has the seven biological replicates of fat body, and the y-axis represents the differential metabolites separated using hierarchical clustering.

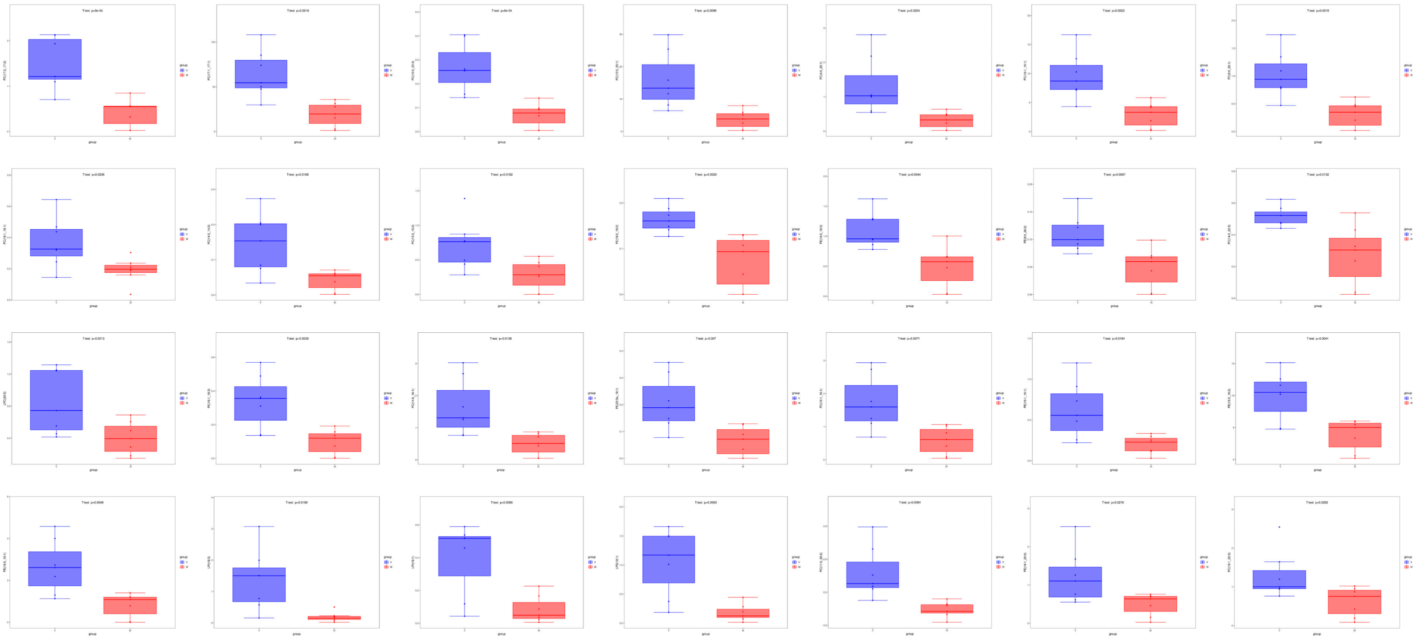

Figure S2. Box plot analysis of 28 metabolites. Most of lipid metabolites were significantly decreased in the fat body of mated bumblebee queens.

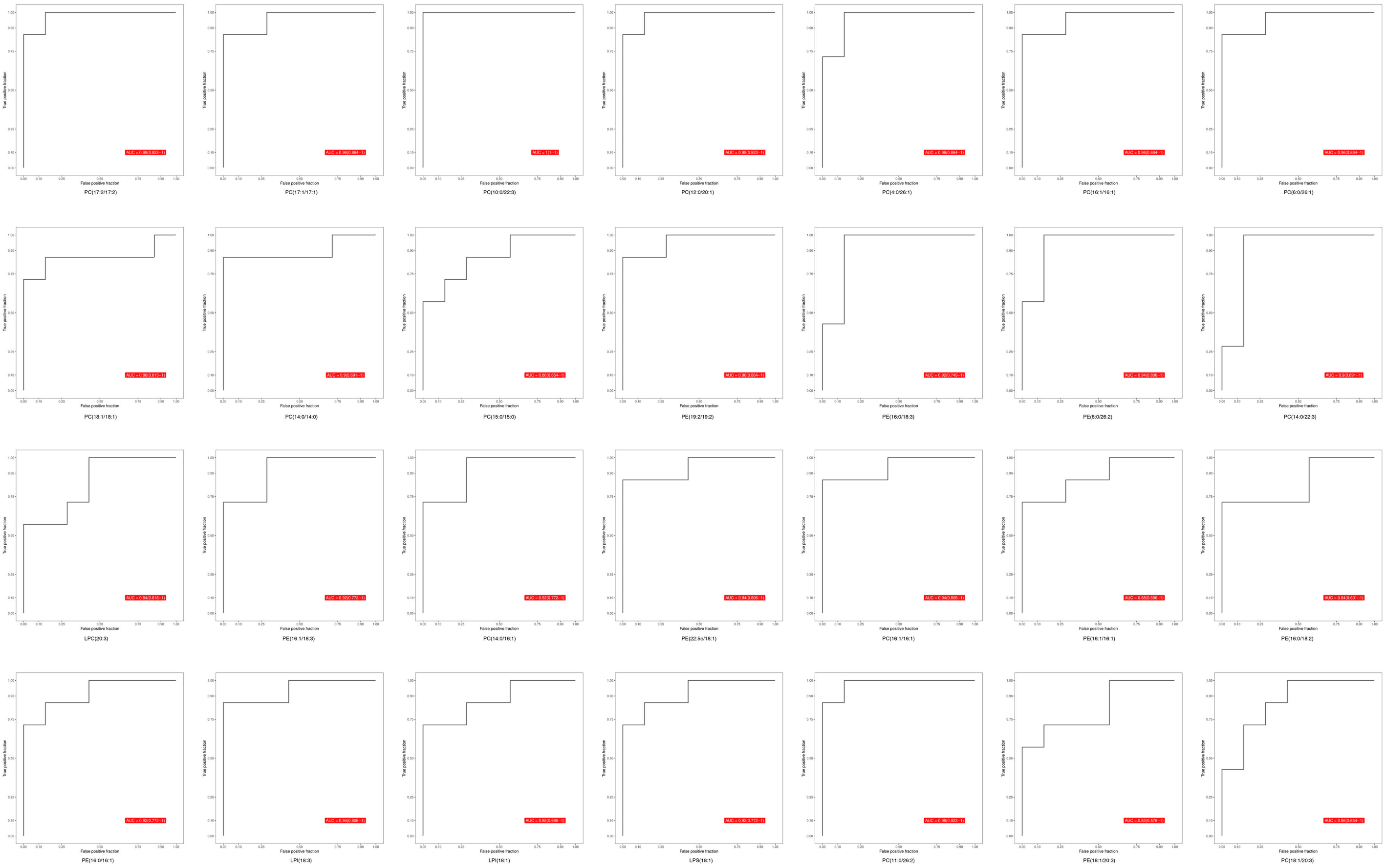

Figure S3. The ROC curve analysis of the 28 metabolites. All of these AUC values are above 0.8, and most are above 0.9, so the accuracy is good.
